# Structural insights into manganese-dependent arylsulfatase from *Enterococcus faecium* and its catalytic promiscuity

**DOI:** 10.1128/mbio.00031-25

**Published:** 2025-08-08

**Authors:** Lulu Guo, Xuanjia Dong, Zetao Hu, Ling Zeng, Zhaohui Jin, Lin Jiang, Wenting Dai, Jinbiao Ma, Shili Chen, Ying Huang

**Affiliations:** 1 Shanghai Key Laboratory of Biliary Tract Disease Research, Department of General Surgery, Xinhua Hospital Affiliated to Shanghai Jiao Tong University School of Medicine, Shanghai, China; 2 State Key Laboratory of Genetic Engineering, Collaborative Innovation Center of Genetics and Development, Department of Biochemistry and Biophysics, Institute of Plant Biology, School of Life Sciences, Fudan University, Shanghai, China; Columbia University, New York, New York, USA

**Keywords:** arylsulfatase, catalytic promiscuity, crystal structure, *Enterococcus faecium*, biomolecule, gut microbiome

## Abstract

Catalytic promiscuity, wherein enzymes catalyze multiple distinct reactions by stabilizing various transition states, is well documented in the alkaline phosphatase superfamily. In this study, we determined the crystal structure of an arylsulfatase from *Enterococcus faecium* (EfAS), revealing a homotetrameric assembly with a windmill-like architecture not observed in other known arylsulfatases or phosphonoester hydrolases. This quaternary structure is stabilized by hydrogen bonding, salt bridges, and hydrophobic interactions, while retaining full accessibility to the catalytic groove. Moreover, by incorporating a manganese ion in its active site, EfAS provides the first crystallographically confirmed example of a Mn²^+^-dependent arylsulfatase, addressing previous uncertainties regarding metal specificity. Functional assays and site-directed mutagenesis showed that EfAS hydrolyzes sulfates, phosphates, and phosphonates, indicating broad substrate specificity. Furthermore, high-performance liquid chromatography–mass spectrometry demonstrated that EfAS removes sulfate groups from key bioactive molecules, such as caerulein and estrone sulfate. Collectively, these findings establish EfAS as an atypical member of the alkaline phosphatase superfamily, featuring a distinct oligomeric organization and broad substrate scope, and suggest its potential role in modulating sulfation of bioactive compounds.

**IMPORTANCE:**

This work provides the first crystallographically confirmed Mn²^+^-dependent arylsulfatase, unveiling a unique “windmill-like” homotetrameric architecture and demonstrating catalytic promiscuity toward sulfates, phosphates, and phosphonates. These findings address longstanding uncertainties about metal specificity in arylsulfatases, highlight the structural and functional diversity of the alkaline phosphatase superfamily, and suggest new strategies for modulating the sulfation of bioactive molecules.

## INTRODUCTION

Enzymes are widely known as specific biological catalysts, yet may exhibit flexibility by catalyzing multiple distinct reactions—a phenomenon known as catalytic promiscuity. Unlike substrate promiscuity, where enzymes act on different substrates without altering the reaction type, catalytic promiscuity involves stabilizing multiple transition states for distinct reactions (1). This flexibility supports adaptability to environmental changes and drives enzyme evolution, especially after gene duplication. Promiscuous enzymes are promising candidates for new catalytic functions (1–5) and are common in the alkaline phosphatase superfamily (6–9).

The alkaline phosphatase superfamily is a diverse class of metalloenzymes with fundamental members, including the alkaline phosphatase (AP), phosphonate monoester hydrolase (PMH), nucleotide pyrophosphatase/phosphodiesterase (NPP), cofactor-independent phosphoglycerate mutase (iPGM), phosphopentomutase (PPM), and a large number of sulfatases represented by arylsulfatase (AS) (10). These enzymes catalyze a broad range of reactions, including phosphoryl group transfer and the hydrolysis of phosphate monoesters/diesters, phosphonate monoesters/diesters, and sulfates. Some members of this superfamily exhibit varying degrees of catalytic promiscuity, while sharing a similar metal coordination architecture and conserved active-site residues, despite limited sequence similarity (10–12). Catalytic activity in these enzymes depends on the presence of specific metal ions. For example, PMH requires one metal ion (commonly Mg²^+^, Mn²^+^, Zn²^+^, or Ca²^+^), whereas arylsulfatases typically require a single metal ion, such as Ca²^+^ or Mg²^+^ (13).

Arylsulfatases, a subset of sulfatases, catalyze the hydrolysis of aryl sulfate esters to produce aryl compounds and inorganic sulfate. These enzymes are widely distributed in nature and enable the hydrolysis of sulfate ester bonds from diverse substrates, including glycosaminoglycans and steroid sulfates. Structural studies of arylsulfatases have revealed a conserved α/β-fold topology characterized by a highly conserved N-terminal catalytic domain and a more variable C-terminal region. Their catalytic centers exhibit significant structural homology, typically featuring a divalent metal ion, such as Ca²^+^ or Mg²^+^, coordinated by polar amino acids arranged in a conserved C/S-X-P/A-X-R motif (X representing any amino acid) (14, 15). Additionally, a proximal LTG motif is essential for substrate binding and catalysis, further emphasizing the critical role of these conserved motifs in enzymatic function (14, 15). While sulfate coordination within arylsulfatase active centers is highly conserved, substantial variability in carbohydrate-binding regions indicates that distinct subfamilies are specifically adapted to different sulfated glycan substrates (16). Catalytic promiscuity has also been documented in arylsulfatases, including those from *Pseudomonas aeruginosa* (PAS) (17) and *Silicibacter pomeroyi* (7).

In this study, we determined the crystal structure of an arylsulfatase from *Enterococcus faecium* (EfAS), which is classified within the S1 family in the SulfAtlas database based on sequence homology (18). The structure revealed a unique windmill-shaped tetramer featuring key residues essential for substrate binding, catalysis, and coordination of divalent metal ions. Notably, EfAS uniquely incorporates a Mn²^+^ ion, marking it as the first arylsulfatase structure identified with this metal core. Structural comparisons indicate a close evolutionary relationship with other members of the alkaline phosphatase family. Functional analyses, including mutagenesis, enzyme kinetics, and mass spectrometry, demonstrated that EfAS exhibits catalytic promiscuity, functioning as both a sulfatase and a phosphatase. Specifically, EfAS was shown to remove sulfate groups from biomolecules, such as caerulein and estrone sulfate, highlighting its versatile catalytic capabilities and potential roles in diverse biological processes.

## RESULTS

### Structural assembly of the windmill-shaped EfAS tetramer

To investigate the structural characteristics of EfAS, we first expressed and purified the recombinant protein. EfAS, fused to an N-terminal His-SUMO tag, was overexpressed in *Escherichia coli* and initially purified using nickel affinity chromatography. The His-SUMO tag was then cleaved by Ulp1 protease, and the protein underwent further purification via anion exchange chromatography and size-exclusion chromatography (Fig. S1).

**Fig 1:**
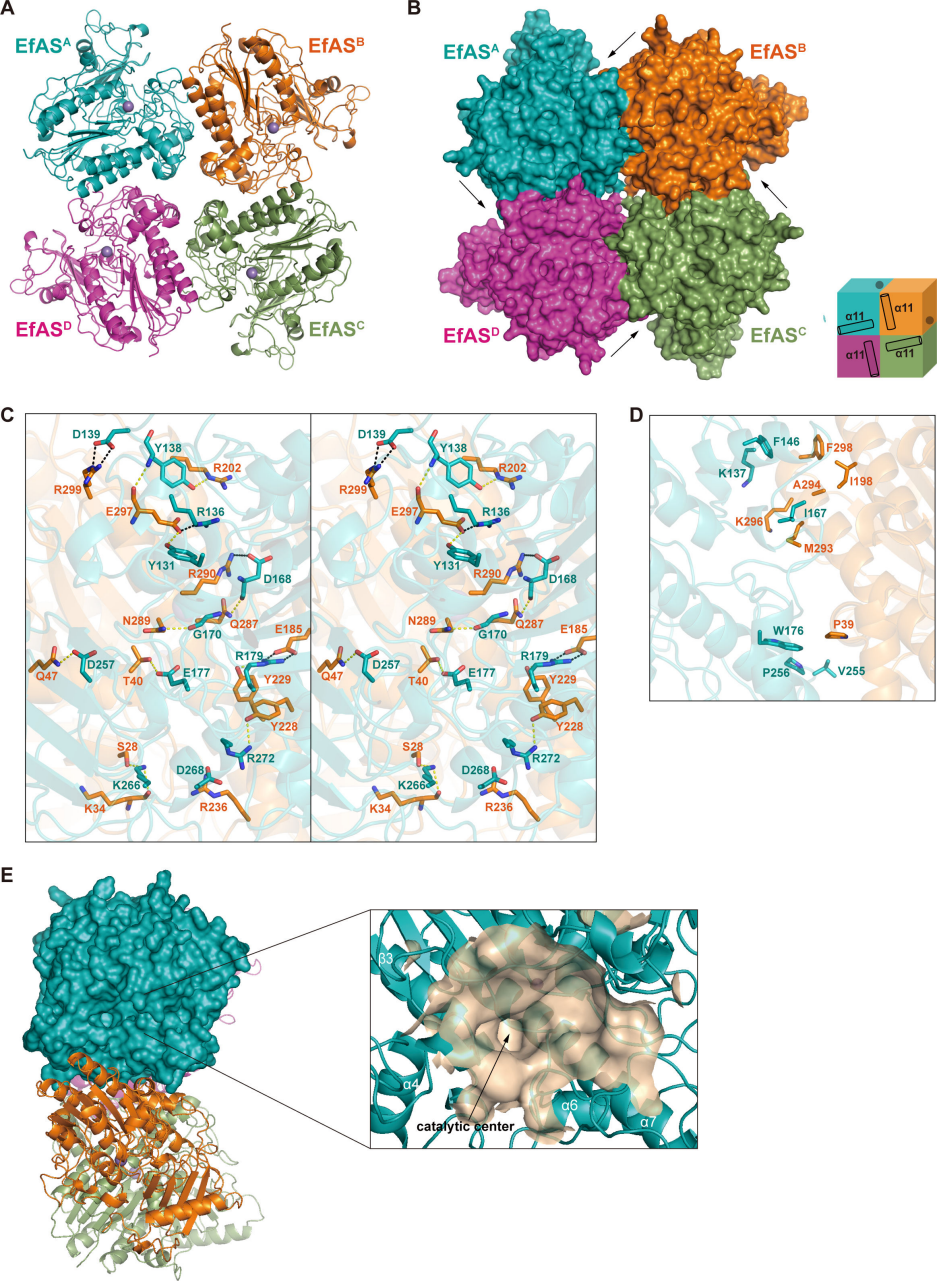
Architecture of EfAS homotetramer. (**A**) Cartoon representation of the EfAS homotetramer with the metal ions shown as spheres. Monomers A, B, C, and D are colored in teal, orange, magenta, green, and magenta, respectively. (**B**) The surface representation of the EfAS tetramer. Arrows indicate the location of the active pockets. The oligomeric structure is shown schematically on the right. Each monomer is shown as a cube. The gray dot in each of the cubes indicates the position of the active site. (**C**) The cross-eye view shows the detailed interactions between EfAS^A^ and EfAS^B^. Hydrogen bonds are shown in yellow dashed lines, while the salt bonds are in black. (**D**) Detailed hydrophobic interactions between EfAS^A^ and EfAS^B^. (**E**) Surface representation showing the catalytic cavity of EfAS^A^, which is still accessible.

The crystal structure of EfAS was determined at 1.96  Å resolution in the P42_1_2 space group, with two crystallographically independent molecules in the asymmetric unit. Structural analysis revealed that EfAS forms a homotetramer through symmetry-related operations, comprising four identical subunits arranged around a central fourfold rotational axis—resembling a windmill assembly (Fig. 1A and B). This quaternary structure is further supported by size-exclusion chromatography, which displays a single monodisperse peak with an estimated molecular weight of 323  kDa (Fig. S1B and C). The interface area between subunits EfAS^A^ and EfAS^B^ is 1331.5  Å², and the Complexation Significance Score (CSS) reaches 0.999, indicating a stable tetramer as evaluated by PISA.

The formation of the homotetramer is primarily mediated by interactions between loops and α-helices of adjacent subunits, involving multiple hydrogen bonds, salt bridges, and hydrophobic interactions (Fig. 1C and D). For example, at the interface between subunits EfAS^A^ and EfAS^B^, 11 hydrogen bonds and five salt bridges play a critical role in ensuring structural integrity (Fig. 1C). Notably, the amide nitrogen of Y138^A^ forms a hydrogen bond with the carbonyl oxygen of E297^B^, while the hydroxyl group of Y138^A^ engages with the Nε of the guanidinium group of R202^B^. The carboxyl group of E297^B^ also forms a hydrogen bond with the hydroxyl group of Y131^A^, reinforcing this interface. Additional hydrogen bonds are established between the main-chain atoms of D168^A^ and G170^A^ and the side-chain amide nitrogens of Q287^B^ and N289^B^, respectively. The side-chain carboxyl groups of E177^A^ and D257^A^ form hydrogen bonds with the hydroxyl group of T40^B^ and the side-chain amide of Q47^B^, respectively. Furthermore, the guanidinium group of R179^A^ interacts with the hydroxyl group of Y229^B^, stabilizing the interface. Finally, the terminal amino group of K266^A^ forms hydrogen bonds with the hydroxyl group of S28^B^ and the main-chain carbonyl of K34^B^. In a similar manner, the guanidinium group of R272^A^ establishes a hydrogen bond with the hydroxyl group of Y228^B^.

Salt bridges further strengthen the interface through electrostatic interactions. Specifically, the carboxyl group of D139^A^ pairs with the guanidinium group of R299^B^, while R136^A^ forms a salt bridge with E297^B^. Similarly, D168^A^ engages with R290^B^, R179^A^ interacts with E185^B^, and D268^A^ pairs with R236^B^. These salt bridges work in concert with the hydrogen bonds to ensure robust stabilization of the homotetramer.

Hydrophobic interactions also contribute to the stability of adjacent subunits. Two distinct hydrophobic clusters are formed at the interface (Fig. 1D). In one cluster, residues K137, F146, and I167 from EfAS^A^ interact with the nonpolar side chains of I198, M193, A194, K296, and F298 from EfAS^B^. In the other cluster, W176, V255, and P256 from EfAS^A^ engage in hydrophobic interactions with P39 from EfAS^B^. These hydrophobic clusters enhance packing at the interface, complementing the hydrogen bonds and salt bridges in stabilizing the tetrameric assembly. Due to the fourfold rotational symmetry, equivalent interactions are present at the subunits B/C and A/D interfaces, mirroring those described for subunits A/B.

The catalytic center of EfAS is located within a groove on the protein’s surface, positioned near the interface between two adjacent subunits (Fig. 1B). This groove forms a deep, wide cavity that remains readily accessible to the solvent (Fig. 1E).

### The overall structure and active site of EfAS

**Fig 2:**
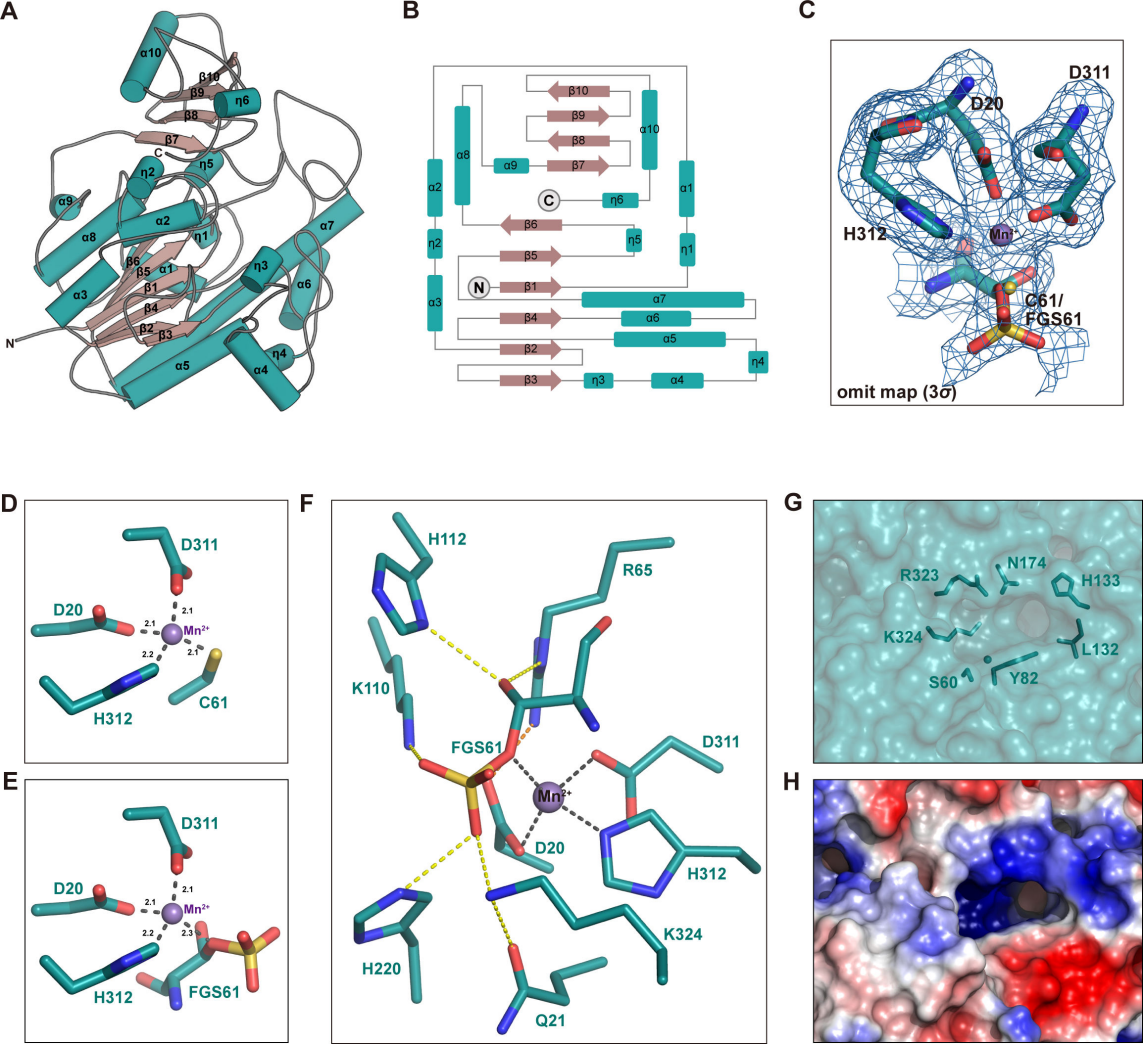
Overall structure of EfAS. (**A**) Cartoon representation of the EfAS crystal structure. Secondary structure elements are color-coded, with β-sheets shown in salmon and α-helices in teal. (**B**) Topology diagram of EfAS, where arrows and rectangles represent β-strands and α-helices, respectively. The color code is as in (**A**). (**C**) Omit map contoured at 3.0 σ, highlighting Mn²^+^ coordination with residues D20, C61/FGS61, D311, and H312. (**D–F**) Structural views of the EfAS active site: (**D**) unreacted state; (**E**) intermediate state after reaction with SO₄²⁻; and (**F**) interactions between FGS61 and surrounding residues. (**G**) Key residues forming the outer boundary of the substrate-binding pocket shown in stick representation. EfAS is shown in transparent surface mode. (**H**) The electrostatic potential surface of EfAS, where red indicates negative and blue positive charges.

The structure was refined to a final *R*_work_ and *R*_free_ of 0.1566 and 0.1766, respectively, comprising residues 9–488 (Table 1). EfAS exhibits a characteristic AP superfamily α/β-fold structure (6), featuring two β-sheet domains: an N-terminal six-stranded (β1–β6) and a C-terminal four-stranded (β7–β10) β-sheet, encompassed by ten α-helices (α1–α10) (Fig. 2A and B). A prominent α7 helix (residues 267–298, 31 amino acids) runs parallel to the N-terminal β-sheet, contributing to tetramer formation (Fig. 2A).

**TABLE 1:** Data collection and refinement statistics

Data collection	EfAS (PDB 9KOQ)
Wavelength (Å)	0.97853
Space group	P42_1_2
Cell dimensions	
a, b, c (Å)	151.6, 151.6, 97.2
*α*, *β*, *γ* (°)	90, 90, 90
Resolution (Å)^*a*^	75.8–1.96 (2.06–1.96)
R_merge_ (%)	0.169 (1.527)
*I*/σ*I*	16.4 (2.7)
Completeness (%)	100 (100)
Redundancy^*a*^	25.9 (24.3)
Refinement	
Resolution (Å)	46.93–1.96
No. of unique reflections	81,957
R_work_	0.1566
R_free_	0.1766
No. of atoms	8,692
Protein	7,902
Mn²^+^	2
Water	788
Average B factors (Å^2^)	
Protein	29.43
Mn²^+^	26.05
Water	37.49
RMSD	
Bond length (Å)	0.0079
Bond angles (°)	0.9769
Ramachandran analysis (%)	
Favored	97.06
Allowed	2.94
Outliers	0

^*a*^ The numbers in parentheses indicate the highest resolution shell.

The catalytic residue C61, positioned at α3’s N-terminus near the N-terminal β-sheet, belongs to the conserved C/S-X-P/A-X-R motif characteristic of sulfatases. This residue undergoes essential posttranslational oxidation of its thiol group to Cα-formylglycine (FGly, 2-amino-3-oxopropionic acid), which is subsequently activated by water to hydroxyformylglycine (HFG), functioning as an aldehyde hydrate (FGH) with two geminal hydroxyl groups (Fig. S2). In our crystal structure, C61 displays dual conformations: unmodified C61 with its –SH group coordinating Mn²^+^ (Fig. 2C and D), and HFG61 covalently bound to sulfate, forming a sulfate-ester intermediate (FGS) (Fig. 2C and E), likely resulting from crystallization in the presence of p-nitrophenyl sulfate.

Metal coordination analysis revealed that optimal electron density maps and B-factors were observed exclusively with Mn²^+^, not Ca²^+^ or Mg²^+^, which correlates with subsequent activity assays showing Mn²^+^-specific catalysis (Fig. 2C). The active site architecture features positively charged residues K110, K324, and R65, which may neutralize carboxylate and sulfate moieties (Fig. 2F). Additional stabilizing interactions include hydrogen bonds between H112’s imidazole ring and FGS61’s hydroxyl group, H220’s nitrogen and FGS61’s sulfate oxygen, and Q21’s hydrogen bond with K324 near FGS61, facilitating substrate binding (Fig. 2F).

The substrate-binding pocket of EfAS exhibits an elongated and narrow shape (Fig. 1E), delineated by residues S60, Y82, L132, H133, N174, R323, and K324, which form the outer boundary of the pocket (Fig. 2G). This spatial arrangement creates a distinct structural environment, characterized by a positively charged interior and a hydrophobic entrance, optimized for binding negatively charged sulfate/phosphate moieties and hydrophobic aryl groups, respectively (Fig. 2H). Such a unique combination of polar and nonpolar residues within and around the pocket likely facilitates interactions with structurally diverse substrates, thus contributing to the enzyme’s substrate promiscuity and catalytic versatility.

### Phylogenetic analysis reveals the distinct evolutionary position of EfAS

**Fig 3:**
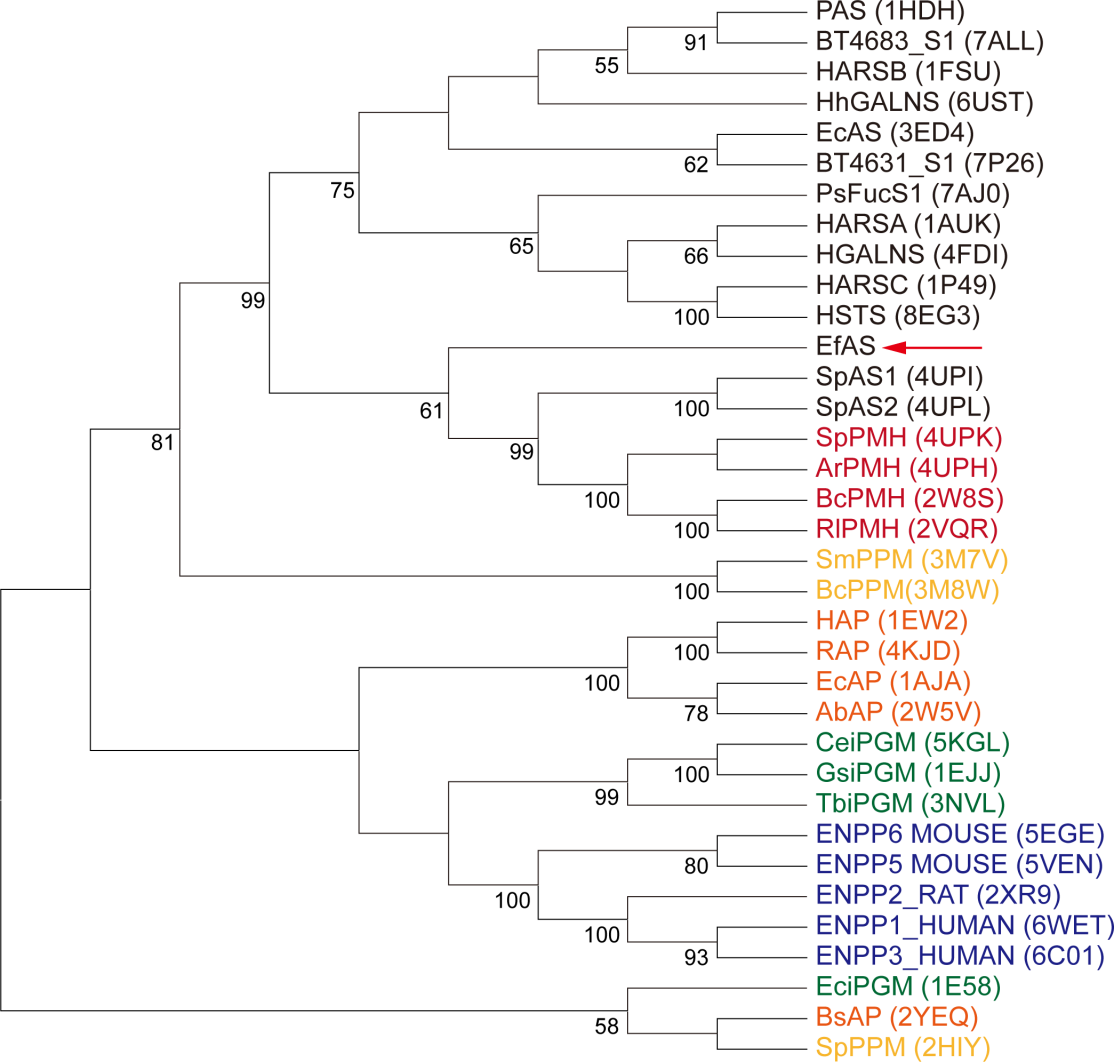
Phylogenetic analysis of the alkaline phosphatase superfamily. The phylogenetic tree of EfAS and members of the alkaline phosphatase superfamily was constructed from MUSCLE multiple alignments of sequences obtained from the UniProt database, including structurally characterized members listed in Table S1. Using MEGA version 11.0.13, the evolutionary history was inferred via the neighbor-joining method, with positions having less than 80% site coverage omitted. Bootstrap values (1,000 replicates) above 50% are displayed next to branches. Subfamilies are color-coded: AS in black, PMH in red, PPM in yellow, AP in orange, iPGM in green, and NPP in blue. The red arrow highlights EfAS.

EfAS belongs to the S1 family of sulfatases according to the SulfAtlas database classification system (18). Although manganese has been reported to occupy the active sites of other sulfatases in minor proportions, it has not been observed as the predominant divalent metal ion. Furthermore, in EfAS, manganese predominates as the major metal ion at the active site. The structure of EfAS exhibits the characteristic AP superfamily fold, featuring a central β-sheet surrounded by α-helices. To investigate its evolutionary context, we conducted a phylogenetic analysis using the neighbor-joining method, comparing EfAS with structurally characterized members of the alkaline phosphatase superfamily (Fig. 3). The resulting phylogenetic tree, supported by bootstrap values ≥ 50%, revealed that EfAS is evolutionarily distinct from conventional arylsulfatases. Instead, it clusters with phosphonoester hydrolases, forming a clade that includes the recently reported catalytically promiscuous SpAS1 and SpAS2 (7). This phylogenetic separation from traditional arylsulfatases is particularly robust, supported by a 99% bootstrap value, highlighting EfAS’s unique evolutionary position within the sulfatase family.

### Distinctive structural features and oligomeric assembly of EfAS

**Fig 4:**
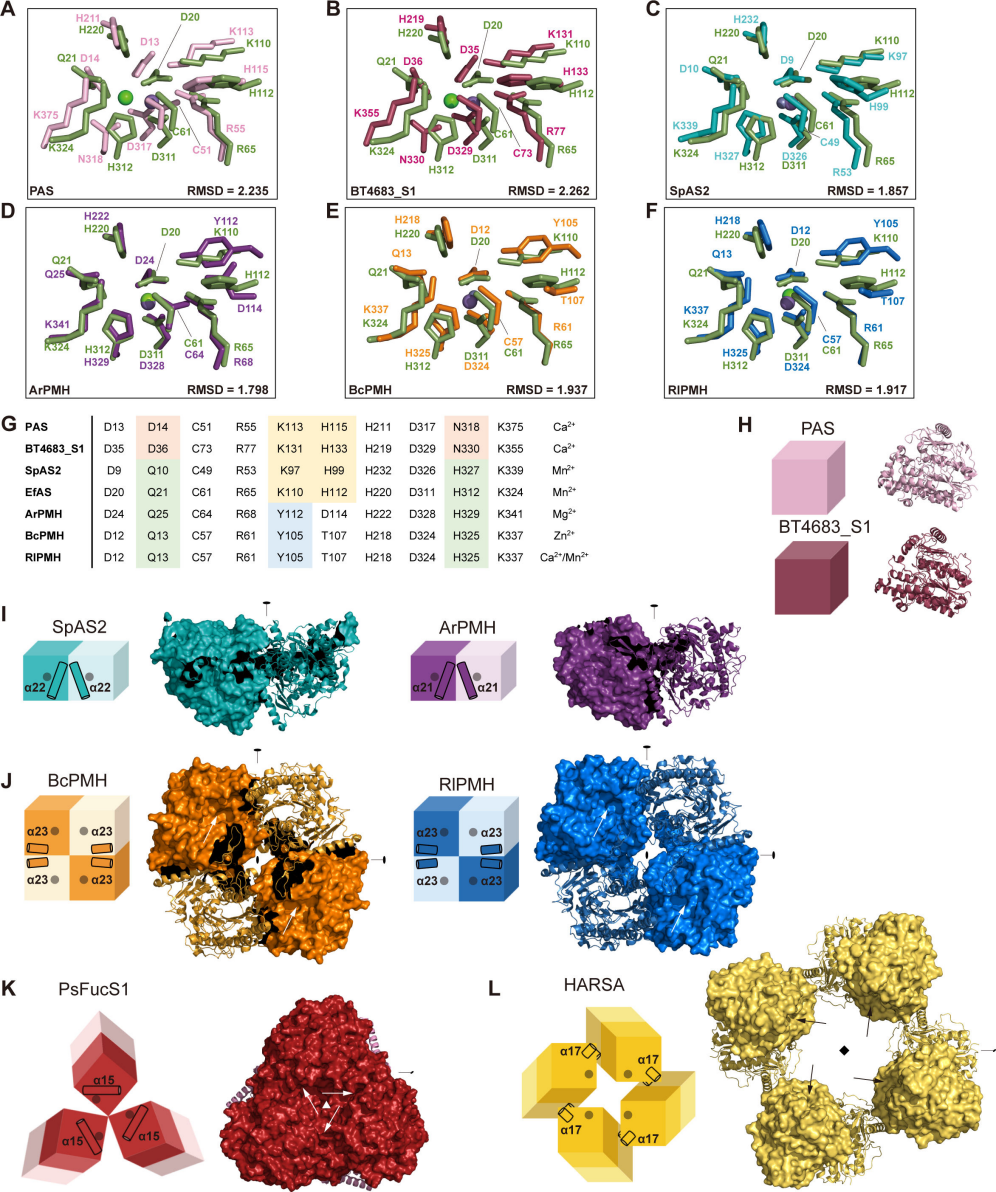
Structural comparison of EfAS and members of the alkaline phosphatase superfamily. (**A–F**) Structural alignment of the active sites of arylsulfatases and phosphonate monoester hydrolases, shown in the same orientation based on a multiple structural alignment. Structures included are PAS (PDB ID 1HDH), BT4683_S1 (PDB ID 7ALL), SpAS2 (PDB ID 4UPL), ArPMH (PDB ID 4UPH), BcPMH (PDB ID 2W8S), and RlPMH (PDB ID 2VQR). (**G**) Table listing the active site residues that distinguish classical ASs from PMHs, with residues color-coded to indicate similarity across different enzymes. (**H-L**) Illustrations of the promoter assemblies in various AP superfamily members: (**H**) monomeric PAS and BT4683_S1, (**I**) dimeric SpAS2 and ArPMH, (**J**) tetrameric BcPMH and RlPMH, (**K**) hexameric PsFucS1 (PDB ID 7AJ0), and (**L**) octameric HARSA (PDB ID 1AUK). I, J, and L highlight the main helices involved in the assembly.

Structural analysis reveals distinctive features of EfAS within the AS/PMH superfamily. While the active site maintains core features of divalent metal coordination through two aspartates (D20 and D311) and the catalytic cysteine (C61), EfAS shares the distinctive fourth coordinating histidine (H312) with PMHs and SpAS2, diverging from the asparagine found in classical ASs (Fig. 4A through G). EfAS also exhibits unique hydrogen-bonding patterns, with lysine (K324) bonding to glutamine (Q21) instead of the classical lysine-aspartate interaction.

Monomeric EfAS shows closer structural similarity to PMHs and SpAS2 (RMSDs 1.798–1.937 Å) than to classical ASs (RMSDs > 2 Å). However, its quaternary structure is notably distinct, featuring a unique homotetrameric assembly with cyclic (C4) symmetry (Fig. 1). This assembly is mediated primarily through interactions between the long α7 helix near the N-terminal β-sheet and loops from adjacent subunits (Fig. 1A), a configuration unprecedented among known AS and PMH structures (Fig. 4H through L).

The oligomeric diversity within the superfamily is substantial: PAS and BT4683_S1 exist as monomers with C1 symmetry (Fig. 4H), while ArPMH and SpAS2 form C2-symmetric dimers (Fig. 4I). Tetrameric structures like BcPMH and RlPMH exhibit D2 symmetry (Fig. 4J), and higher-order assemblies include a D3-symmetric PsFucS1 hexamer (Fig. 4K) and D4-symmetric HARSA octamer (Fig. 4L). These assemblies typically involve distinct interface types, with larger contacts formed through C-terminal β-sheet-spanning α-helices and smaller supporting interfaces.

Higher-order assemblies demonstrate diverse interaction mechanisms. HARSA forms its octameric structure through C-terminal β-sheet-spanning α-helices, while PsFucS1 achieves hexameric assembly through N-terminal α-helical bundles in a trimeric ring configuration. Notably, while α15 in EfAS corresponds to the C-terminal β-sheet-spanning helix typically involved in oligomerization, it does not participate in EfAS’s tetrameric assembly, further emphasizing its structural uniqueness within the superfamily.

### Catalytic efficiency and substrate preference of EfAS

**Fig 5:**
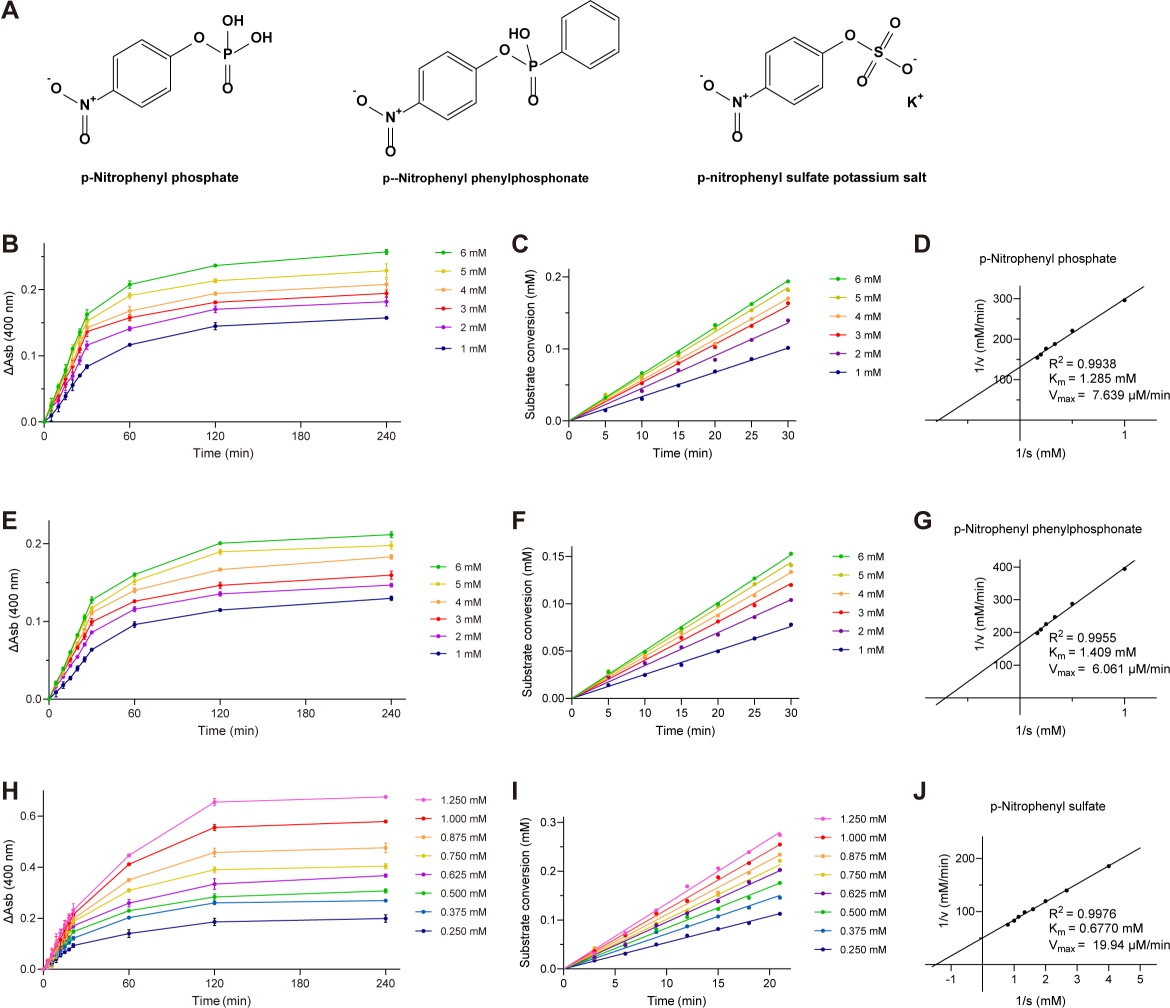
Kinetic analysis of EfAS with different substrates. (**A**) Chemical structures of the three selected substrates. (**B**) Kinetics of EfAS assessed using the p-nitrophenol colorimetric assay, monitoring absorbance at 400 nm over time with various p-nitrophenyl phosphate concentrations in the presence of EfAS. (**C**) Linear initial kinetics of EfAS-catalyzed p-nitrophenyl phosphate in the first 30 minutes. (**D**) Nonlinear regression of EfAS activity with p-nitrophenyl phosphate. Initial velocity (Vi, μM/min) was estimated by GraphPad Prism software (9.5.0) fitting data to the Michaelis-Menten model. Error bars represent standard deviation from triplicate measurements at each substrate concentration. (**E–G**) Similar analyses as in (**B–D**) performed with p-nitrophenyl phenylphosphonate. (**H–J**) Similar analyses as in (**B–D**) performed with p-nitrophenyl sulfate, measuring Vi during the first 21 minutes.

Phylogenetic and structural analyses prompted an investigation into the catalytic promiscuity of EfAS. To this end, we tested three substrates representing major hydrolysis types in the AP superfamily: p-nitrophenyl phosphate (monophosphate), p-nitrophenyl phosphonate (monophosphonate), and p-nitrophenyl sulfate (monosulfate) (Fig. 5A). Catalytic activity was assessed using the p-nitrophenol (pNP) colorimetric method at 400 nm, based on pNP’s distinct UV absorption peak at 400 nm under alkaline conditions (Fig. S3). Absorbance measurements enabled precise quantification of p-nitrophenol release and enzymatic activity towards each substrate. Initial characterization examined environmental factors influencing EfAS activity. The enzyme displayed tolerance to acidic conditions, with an optimal pH of 4.5 (Fig. S4A through C) and maximal catalytic performance at 52°C and pH 4.5 (Fig. S4D through F). Supplementation with 1 mM MnCl_2_ further enhanced activity at 37°C (Fig. S4G through I).

Under the optimized conditions of 37°C, pH 4.5, and 1 mM MnCl_2_, all three substrates exhibited Michaelis-Menten kinetics, showing linear initial reaction rates with increasing substrate concentrations (Fig. 5B through J). Kinetic parameters, including the Michaelis constant (*K*_*m*_), catalytic rate constant (*k*_cat_), and catalytic efficiency (*k*_cat_/*K*_*m*_), were determined for each substrate (Table 2). EfAS demonstrated catalytic efficiency for all substrates, but its activity towards the monosulfate substrate was highest, with *k*_cat_/*K*_*m*_ values approximately five to seven times greater than those for monophosphate substrates. These results align with the hypothesis that EfAS functions primarily as a sulfatase.

**TABLE 2:** Experimental measurements of catalytic promiscuity in EfAS^*a*^

Substrate	Category	Kinetic parameters		
		*K*_*m*_ (×10^−3^ M)	*K*_cat_ (×10^−2^ s^−1^)	*K*_cat_/*K*_*m*_ (M^−1^·s^−1^)
p-nitrophenyl phosphate	Phosphate monoester	1.285	3.183	24.77
p-nitrophenyl phenylphosphonate	Phosphonate monoester	1.409	2.525	17.92
p-nitrophenyl sulfate	Sulfate monoester	0.6770	8.308	122.7

^*a*^ The assay conditions were as follows: 0.05 M sodium acetate buffer (pH 4.5), 1 mM MnCl_2_, at 37°C. The kinetic parameters were determined by plotting 1/V (mM/min) against 1/S (mM) using Lineweaver-Burk plot. The linearity is determined by the *R*^2^ value.

### Validation of key residues involved in EfAS catalytic activity

**Fig 6:**
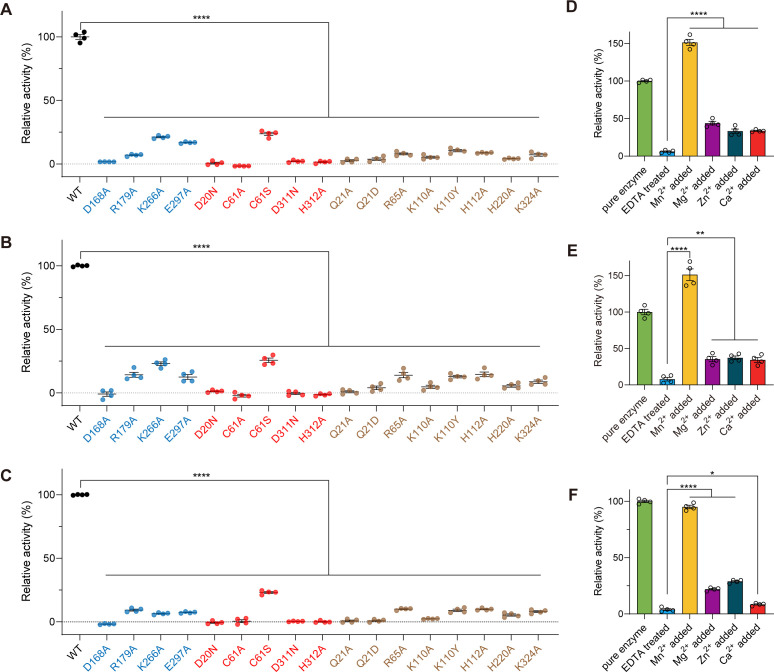
Influence of key residues and metal ions on EfAS catalytic activity. (**A–C**) Enzyme activity of WT and mutant EfAS was measured using the p-nitrophenol colorimetric assay after co-incubation with 2 mM p-nitrophenyl phosphate (**A**), 2 mM p-nitrophenyl phenylphosphonate (**B**), or 1 mM p-nitrophenyl sulfate (**C**) for 30 minutes at pH 4.5 and 37°C. Mutants include Mn²^+^ coordination site variants, catalytic pocket residues, and tetramer-interface residues. Blue bars indicate interface mutants, red bars indicate Mn²^+^ coordination site mutants, and brown bars indicate substrate-binding pocket mutants. (**D–F**) Enzyme activity of WT and EDTA-treated EfAS was measured under the same conditions as above, supplemented with 1 mM of various divalent metal ions, after co-incubation with 2 mM p-nitrophenyl phosphate (**D**), 2 mM p-nitrophenyl phenylphosphonate (**E**), or 1 mM p-nitrophenyl sulfate (**F**).

The crystal structure of EfAS revealed a windmill-shaped tetrameric assembly (Fig. 1A and B). To investigate whether this tetrameric structure is essential for catalytic activity, we performed site-directed mutagenesis targeting residues identified as critical for tetramer formation based on structural analysis (Fig. 1C). Specifically, we generated four single mutants: D168A, R179A, K266A, and E297A (Fig. S5A). Size-exclusion chromatography revealed that all mutants eluted at significantly later volumes compared to the wild-type protein, confirming disruption of the tetrameric assembly and indicating their monomeric status (Fig. S5B). Catalytic assays further demonstrated that all four mutants exhibited dramatically reduced or completely abolished enzymatic activity (Fig. 6A through C). These results highlight that the tetrameric assembly plays a critical role in maintaining the catalytic function of EfAS.

The crystal structure of EfAS revealed that C61 is directly involved in coordinating Mn²^+^, a feature crucial to its catalytic function (Fig. 2D through F). Consistently, activity tests showed that supplementation with 1 mM Mn²^+^ significantly enhanced enzymatic activity, whereas other divalent metal ions, including Mg²^+^, Zn²^+^, and Ca²^+^, had negligible effects (Fig. S4G through I). To confirm the importance of Mn²^+^ coordination and the role of key residues in the catalytic center, five single-point mutations were introduced: D20N, C61A, C61S, D311N, and H312A. Activity assays demonstrated that D20N, C61A, D311N, and H312A mutants lost catalytic activity completely, displaying no substrate conversion across all three substrates tested (Fig. 6A through C). Notably, the C61S mutant retained approximately 20–35% of residual catalytic activity, likely because serine can also be oxidized to formylglycine, albeit less efficiently than cysteine. These findings underscore the essential role of Mn²^+^ and the catalytic center residues in EfAS function, establishing EfAS as a Mn²^+^-dependent sulfatase.

To further validate the Mn²^+^ dependence and eliminate the potential influence of metal ions carried over from protein purification, wild-type EfAS was treated with high concentrations of EDTA to chelate and remove any bound metal ions, followed by extensive dialysis. EDTA treatment entirely abolished the hydrolytic activity of EfAS (Fig. 6D through F). Subsequent reactivation experiments with various divalent ions revealed that Mn²^+^ fully restored and enhanced catalytic activity, whereas other ions, including Ca²^+^ (common in some other sulfatases), provided only minimal reactivation (Fig. 6D through F). Additionally, to validate the functional importance of residues shaping the substrate-binding pocket, eight single-point mutants were generated, which are Q21A, Q21D, R65A, K110A, K110Y, H112A, H220A, and K324A. Activity assays showed that all mutants exhibited dramatically reduced or completely abolished catalytic activity (Fig. 6A through C). Q21A, Q21D, and K110A resulted in complete loss of function, while R65A, K110Y, H112A, H220A, and K324A retained only minimal residual activity. Collectively, our results demonstrate that tetrameric assembly, Mn²^+^ coordination, and substrate pocket architecture are all essential for EfAS activity.

### EfAS catalyzes bioactive molecule desulfation

To investigate the ability of EfAS to catalyze the removal of sulfonic groups from biologically active molecules, we selected two substrates: caerulein and estrone sulfate. Both molecules are clinically and physiologically relevant because their biological functions are closely linked to their sulfation modifications. Caerulein, a decapeptide and potent cholecystokinin (CCK) receptor agonist, plays a critical role in gastrointestinal physiology. Removal of its sulfation, particularly from the sulfated tyrosine residue, significantly reduces its affinity for CCK receptors, including those in the pancreas (19). Desulfated caerulein, for instance, cannot induce gastric acid secretion in animal models of acute pancreatitis (20). Estrone sulfate, on the other hand, is an inactive form of estrone that, upon desulfation, is converted into active estrone, a molecule implicated in various physiological processes. Elevated levels of estrone sulfate, along with estrone, have a positive correlation with increased risk of breast cancer in both premenopausal and postmenopausal women (21–23). These biologically active molecules were selected because of their functional dependence on sulfation and their potential relevance to disease pathogenesis.

Recent studies have identified *Enterococcus faecium*, the species that encodes EfAS, as an enriched member of the tumor-associated microbiota in patients with gallbladder cancer (GBC) (24). This bacterial enrichment has raised questions about its potential contribution to the oncogenic microenvironment. Thus, exploring the enzymatic activity of EfAS on biomolecules like caerulein and estrone sulfate is of particular significance, as it may reveal links between microbial metabolism, disease pathogenicity, and therapeutic applications.

**Fig 7:**
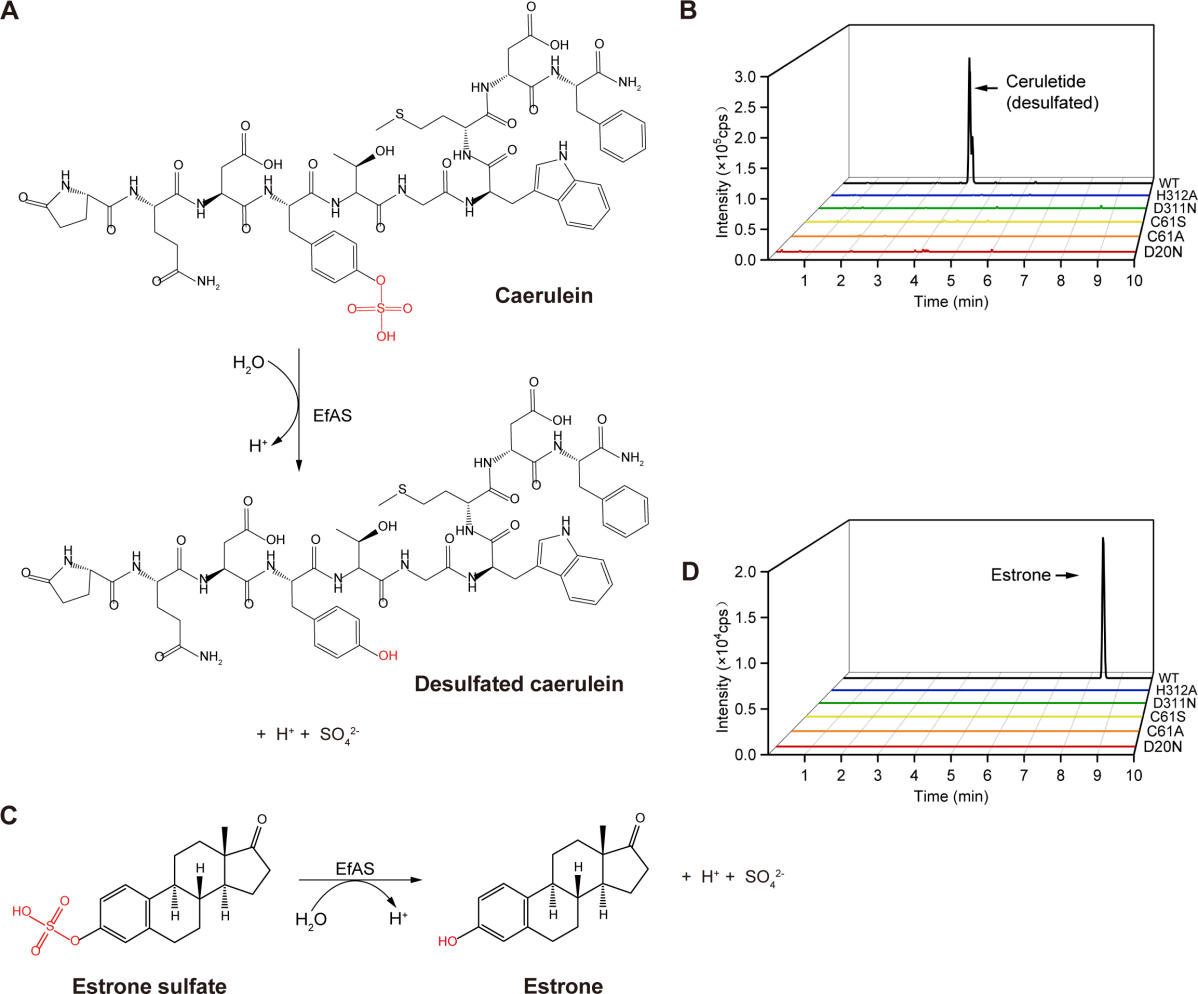
EfAS can catalyze the desulfonation of caerulein and estrone sulfate. (**A**) Diagram illustrating the catalytic reaction of EfAS converting caerulein to desulfated caerulein. (**B**) HPLC-LC/MS chromatography profiles demonstrated that WT partially hydrolyzed caerulein after 12 hours. (**C**) Diagram illustrating the catalytic reaction of EfAS converting estrone sulfate to estrone. (**D**) HPLC-LC/MS chromatography profiles demonstrate that WT partially hydrolyzed estrone after 12 hours.

To determine EfAS activity on these biomolecules, high-performance liquid chromatography–mass spectrometry (HPLC-MS) was employed to detect desulfation products. Under optimized reaction conditions (37°C, pH 4.5, and 1 mM MnCl₂), wild-type EfAS and its catalytic mutants were incubated with caerulein and estrone sulfate for 12 hours, respectively, followed by detection of reaction products. Upon incubation of wild-type EfAS with caerulein, a desulfated product was observed with a retention time of 4.10 minutes. This product was absent when mutant EfAS (D20N, C61A, D311N, or H312A) was used (Fig. 7A and B). Similarly, incubation of wild-type EfAS with estrone sulfate yielded a desulfated product with a retention time of 8.41 minutes, which was also not detected in reactions with mutant enzymes (Fig. 7C and D). These results demonstrate that wild-type EfAS effectively hydrolyzed and removed sulfate groups from both caerulein and estrone sulfate.

## DISCUSSION

Our study reveals the structural and functional features of EfAS, a Mn²^+^-dependent arylsulfatase from *E. faecium*. It adopts a novel windmill-shaped tetrameric assembly, stabilized by hydrogen bonding, salt bridges, and hydrophobic interactions. Unlike monomeric or dimeric arylsulfatases, this arrangement allows full access to each active site and may contribute to enzyme stability under acidic or elevated temperature conditions. Site-directed mutations at the interface which disrupt tetramer formation abolished catalytic activity, indicating that oligomerization is essential for function.

EfAS incorporates a manganese ion in its active site, marking the first crystallographically resolved Mn²^+^-dependent arylsulfatase. Although previous studies have suggested Mn²^+^ dependence from activity assays, EfAS structure provides direct structural evidence of Mn²^+^ coordination with the catalytic FGS intermediate. Mn²^+^ offers higher Lewis acidity and more flexible coordination geometry than Ca²^+^, facilitating transition state stabilization and bond polarization during nucleophilic attack (25, 26). While the primary metal-binding residues are conserved, EfAS’s preference for Mn²^+^ over Ca²^+^ likely arises from second-shell interactions and microenvironmental tuning. Mutations in those non-conserved residues further support their role in shaping metal specificity and catalytic competence.

Moreover, although EfAS and SpAS2 share nearly identical metal coordination geometry (Fig. 2), their substrate-binding pockets differ substantially in architecture. In SpAS2, a long β7–β8 loop forms an α-helix that partially occludes the active site, while EfAS lacks this helix, resulting in a more open and accessible cleft. Similarly, the β3–α4 region adopts a short anti-parallel β-sheet in SpAS2 but forms a single turn in EfAS, altering pocket shape and accessibility from a different angle. These local conformational features reshape the pocket topology without altering the catalytic center, potentially governing substrate orientation, size tolerance, and access routes. Comparative surface analysis (Fig. S6) highlights these distinctions and suggests that architectural divergence outside the conserved core contributes to substrate recognition and functional adaptation.

Compared to canonical AP superfamily enzymes, which often exhibit 10^2^- to 10^7^-fold higher catalytic efficiency toward their native substrates relative to promiscuous ones, EfAS displays a more balanced activity profile, with less than 10-fold variation across sulfate, phosphate, and phosphonate esters (Table 3). This unusually narrow range suggests that EfAS may be functionally adapted to accommodate a chemically diverse substrate pool rather than being optimized for a single class. Structural features revealed that the substrate-binding pocket of EfAS is chemically heterogeneous, incorporating polar, aromatic, hydrophobic, and charged residues, and exhibits an open geometry. Although differences in surface electrostatics and pocket shape are observed among EfAS, SpAS2, PAS, and BcPMH (Fig. S6), direct correlation with substrate specificity remains unresolved. The combination of structural openness and moderate, nonselective catalytic efficiencies may thus reflect an alternative strategy for substrate recognition, potentially shaped by ecological pressure to utilize variable organosulfur or organophosphate compounds.

**TABLE 3:** Comparison of the catalytic efficiency (*K*_cat_/*K*_*m*_, M^−1^·s^−1^) of the alkaline phosphatase superfamily members across different substrates

Enzyme	Substrate		
	p-nitrophenyl phosphate	p-nitrophenyl phenylphosphonate	p-nitrophenyl sulfate
EfAS	2.5 × 10^1^	1.8 × 10^1^	1.2 × 10^2^
BcPMH (27)	2.2 × 10^1^	1.5 × 10^4^	5.9 × 10^−1^
PAS (7, 17)	8.4 × 10^1^	1.5 × 10^−2^	2.3 × 10^7^
SpAS2 (7)	5.6 × 10^0^	4.0 × 10^2^	2.1 × 10^5^

Phylogenetically, EfAS clusters more closely with phosphonoester hydrolases than with classical arylsulfatases, suggesting an evolutionary trajectory shaped by functional demands for substrate diversification. Its substrate promiscuity, metal flexibility, and tetrameric assembly may represent adaptive responses to complex environmental pressures, including nutrient availability and host interactions. These features parallel multifunctional AP enzymes in marine bacteria, where broad specificity supports ecological resilience (2, 28).

Moreover, *E. faecium* has been identified as an abundant member of the microbiota in gallbladder cancer (24). Our findings reveal that EfAS can hydrolyze sulfate groups from bioactive molecules like caerulein and estrone sulfate, which are significant in gastrointestinal function and endocrine regulation, respectively. Desulfation of these molecules diminishes their biological activity, suggesting that EfAS plays a role in modulating host signaling pathways and potentially influencing disease outcomes. For instance, desulfation of caerulein reduces its cholecystokinin receptor agonistic activity, while the removal of sulfate from estrone sulfate activates estrone, a hormone implicated in breast cancer risk. The capability of EfAS to alter the activity of these bioactive molecules underscores its potential impact on microbial metabolism and host-microbe interactions. This enzymatic activity suggests potential biotechnological applications, such as the regioselective desulfation of pharmaceuticals to modulate their activity profiles. Additionally, understanding EfAS’s action on sulfated compounds can inform therapeutic strategies to manage hormone-related conditions and gastrointestinal disorders.

In conclusion, this study provides mechanistic insights into the structural and functional innovation of EfAS, highlighting its role as a versatile enzyme within the alkaline phosphatase superfamily. The unique tetrameric assembly and broad catalytic scope underscore the potential of EfAS in biotechnological and therapeutic contexts, particularly in the metabolism of sulfated bioactive compounds. Further research into EfAS’s substrate scope and regulatory mechanisms will enhance our understanding of its role in microbial ecology and its application potential, paving the way for novel treatments targeting microbial metabolism and host-microbe interactions in diseases, such as gallbladder cancer and hormone-related disorders.

## MATERIALS AND METHODS

### Chemical reagents

The following reagents were used in this study: p-nitrophenol (Shanghai AbMole Bio-Technology Co., Ltd.), p-nitrophenyl phosphate, p-nitrophenyl phosphonate, p-nitrophenyl sulfate, and estrone sulfate piperazine salt (Shanghai Yuanye Bio-Technology Co., Ltd.). Additionally, caerulein and desulfated caerulein were obtained from MedChemExpress, and estrone was purchased from Shanghai Macklin Bio-Technology Co., Ltd.

### Cloning

A synthetic, *E. coli* codon-optimized version of the *E. faecium* arylsulfatase (EfAS) was cloned into a pSMT3 vector using the BamHI and SalI sites, resulting in an N-terminal Ulp1-cleavable hexahistidine–SUMO-tagged version of the enzyme.

### Protein expression and purification

The hexahistidine–SUMO-tagged EfAS was expressed in *E. coli* BL21 (DE3) cells. Cultures were grown in an LB medium containing 50 mg/L kanamycin at 37°C with shaking at 200 rpm until an optical density at 600 nm (OD₆₀₀) of 0.6 was achieved. Protein expression was induced with 0.2 mM isopropyl-β-D-thiogalactopyranoside (IPTG), followed by incubation at 16°C overnight. Cells were harvested by centrifugation at 5,000 rpm for 10 minutes, lysed at 4°C using a French press (JNBIO), and clarified by ultracentrifugation at 18,000 rpm for one hour at 4°C.

The tagged EfAS was purified by affinity chromatography using a HisTrap HP column (Cytiva). The hexahistidine-SUMO tag was then removed by Ulp1 protease cleavage, and the resulting protein was subjected to ion-exchange chromatography on a HiTrap Q column (Cytiva) and eluted with a NaCl gradient. A final purification step involved size-exclusion chromatography using a HiLoad Superdex 200 pg column (Cytiva) in 20 mM Tris (pH 8.0) and 500 mM NaCl. Fractions containing high-purity EfAS, confirmed by SDS-PAGE, were pooled, concentrated using a 30 kDa cutoff centrifugal concentrator, flash-frozen in liquid nitrogen, and stored at –80°C for subsequent assays.

### Crystallization, data collection, and structure determination

The protein EfAS was purified to a 5 mg/mL concentration and then crystallized using the vapor diffusion method at 17°C. Initial screening and optimization of crystal formation were performed using PEG3350 as a precipitant prepared at 6–11% w/v in 0.1 M sodium cacodylate, pH 6.2–6.5, and supplemented with 0.2 M MnCl_2_. The highest diffraction quality crystals of EfAS were formed by mixing a 5 mg/mL solution at a 1:1 ratio of reservoir solution comprising 0.1 M sodium cacodylate, pH 6.4, 8% PEG3350, and 0.2 M MnCl_2_.

The crystals were rapidly frozen in liquid nitrogen after being transferred to a cryoprotectant solution containing the reservoir solution supplemented with 25% (v/v) glycerol.

X-ray diffraction data were collected at the BL18U1 beamline of the Shanghai Synchrotron Radiation Facility (SSRF) and processed using the HKL3000 software suite (29). The molecular replacement method was employed to solve the structure using the structure generated from AlphaFold 3 (30, 31) as the search model. Model building and refinement were performed iteratively with COOT and Phenix (32). Data collection and refinement statistics are provided in Table 1. The buried surface area was calculated using PISA, and structural visualizations were generated with PyMOL (version 1.8, Schrödinger, LLC) (33). The coordinates have been deposited in the Protein Data Bank under accession code 9KOQ.

### Phylogenetic analysis

The protein sequences were retrieved from the Universal Protein (UniProt) database. The phylogenetic tree from Bihani, Subhash C et al. (10) served as a guide for identifying known AP, NPP, and some sulfatases. The EfAS sequence was queried against the UniProt database using the Protein BLAST online tool. The retrieved protein sequences were aligned using the MUSCLE program with default settings. Phylogenetic trees were constructed using MEGA version 11.0.13 (34). The evolutionary history was inferred using the neighbor-joining method. Bootstrap support values (1,000 replicates) are displayed at the branch nodes. The evolutionary distances were computed using the p-distance method and are in the units of the number of amino acid differences per site.

### Enzyme assays

Enzyme activity assays were performed as previously described (35). All experiments were conducted in triplicate using independently prepared samples. Substrate hydrolysis was assessed using 2 mM para-nitrophenyl phosphate, 2 mM para-nitrophenyl phenylphosphonate, or 1 mM para-nitrophenyl sulfate as substrates. Reactions were carried out in a total volume of 10 µL, containing 4 µM purified enzyme, the indicated substrate concentration, 50 mM buffer at the specified pH, and 1 mM metal ion (or none for control conditions). Enzyme and reagents were pre-equilibrated separately at the desired reaction temperature for 10 minutes before being mixed to initiate the reaction. Following incubation under defined conditions, reactions were terminated by the addition of 2.5 µL of 5 M NaOH. Absorbance was measured at 400 nm using a μDrop plate, and the concentration of para-nitrophenol was quantified using a standard calibration curve.

To assess the effect of pH on enzymatic activity, reactions were performed at 37°C in buffers spanning pH 3.6–9.0: sodium acetate (pH 3.6–5.0), MES (pH 5.5–7.0), and Tris-HCl (pH 7.5–9.0), all containing 1 mM Mn²^+^. After a 30-minute incubation, reactions were terminated, and the amount of pNP released was quantified. Activity was normalized to the maximum observed activity (set as 100%). Temperature dependence was evaluated in Mn²^+^-containing buffer at pH 4.5 over a temperature range of 12–67°C in 5°C increments. Relative activities were calculated by normalizing to the activity at the optimal temperature.

The influence of metal ions was examined at pH 4.5 and 37°C by supplementing the reaction buffer with 1 mM Ca²^+^, Mg²^+^, Zn²^+^, Mn²^+^, or no added metal. Enzymatic activity was expressed relative to the metal-free control (defined as 100%). Kinetic parameters were determined under optimal conditions (pH 4.5, 1 mM Mn²^+^, and 37°C) using a range of substrate concentrations. Initial reaction rates were obtained from the linear phase of product formation, and *K*_*m*_ and *V*_max_ values were calculated using Lineweaver–Burk double-reciprocal plots.

### Metal ion removal and activity reconstitution in EfAS

To remove metal ions, wild-type EfAS was diluted to a concentration of 10 mg/mL and incubated with 25 mM EDTA in a buffer solution in 1 mL Eppendorf tubes at 4°C with gentle agitation overnight or longer. Following EDTA treatment, the proteins were dialyzed at 4°C for 24 hours against a metal-free buffer of 20 mM Tris, pH 8.0, and 500 mM NaCl. Dialysis was performed using MD77 dialysis tubing (MW cutoff 14,000), with the buffer solution changed thrice during dialysis. Post-dialysis, the proteins were further exchanged into fresh metal-free buffers of the same composition. For metal reconstitution, 1 mM of MnCl₂, CaCl₂, MgCl₂, or ZnCl₂ was added directly to the EfAS on ice. The resulting mixtures were incubated for 1 hour at 4°C to facilitate metal ion binding.

### EfAS reaction monitoring by HPLC/MS

To assess EfAS activity, 50 nM caerulein or estrone sulfate was incubated with purified proteins (wild type or mutants) at 25 nM in 0.05 M Na-acetate buffer (pH 4.5) at 37°C. The reaction mixture was incubated overnight at 37°C, followed by adding an equal volume of methanol and vacuum drying. Methanol-water (7:3, v/v) was then added to the dry residue for re-dissolution, and centrifugation was performed at 15,000 × *g* for 10 minutes to collect the supernatant. The supernatant was analyzed by ultra-high performance liquid chromatography (UltiMate 3000 UHPLC, Thermo Fisher) coupled with high-resolution quadrupole-orbitrap mass spectrometry (Q-Exactive Plus, Thermo Fisher).

The supernatant was separated on a BEH C8 column (1.7 µm, 2.1 × 100 mm). The column temperature was maintained at 45°C, and the flow rate was 0.35mL/min.

For estrone sulfate and estrone, mobile phase A was 100% water, mobile phase B was 100% acetonitrile, both containing 0.1% formic acid. The gradient started with 5% B and maintained for 2 minutes, then increased linearly to 30% B at 4 minutes, 50% B at 8 minutes, 80% B at 10 minutes, and 100% B at 14 minutes, where it was held for 1 minute. The gradient then decreased to 5% B at 15.1 minutes and was maintained for another 0.9 minute to equilibrate the column. The total gradient time was 16 minutes.

The mass spectrometry was operated in positive/negative ion switching modes. For the positive ion mode, the spray voltage was 3,800 V. For the negative ion mode, the spray voltage was 3,000 V. For both the ion modes, the capillary temperature was 320°C, the aux gas heater temperature was 350°C, the sheath gas flow rate was 35, the aux gas flow rate was 8, and the S-lens RF level was 50. The mass resolution of full-scan MS was 70,000, and the m/z scan range was 100–1,200.

For caerulein and desulfated caerulein, mobile phase A was a 10 mmol/L ammonium bicarbonate aqueous solution, mobile phase B was 100% acetonitrile. The gradient started with 5% B and was maintained for 1 minute, then increased linearly to 100% B at 8 minutes and was maintained for 1 minute. It then decreased to 5% B at 9.1 minutes and was maintained for another 1.9 minutes to equilibrate the column. The total gradient time was 11 minutes.

The mass spectrometry was operated only in negative ion mode. The m/z scan range of full-scan MS was 150–1,500, with the other parameters the same as before.

### Statistical analysis

All assays were carried out in triplicate or quadruplicate, with at least three independent experiments. Statistical analyses were conducted using GraphPad Prism 9, and data are presented as mean ± standard error. Group comparisons were evaluated using one-way ANOVA followed by Tukey’s multiple comparison test. The figures indicate the significance level using the following notations: ∗*P* < 0.05, ∗∗*P* < 0.01, and ∗∗∗*P* < 0.001.

## Data Availability

The coordinates have been deposited in the Protein Data Bank (PDB) under accession number 9KOQ.

## SUPPLEMENTAL MATERIAL

Supplemental Material mbio.00031-25-s0001.pdf Table S1 and Fig. S1 to S6.

ASM does not own the copyrights to Supplemental Material that may be linked to, or accessed through, an article. The authors have granted ASM a non-exclusive, world-wide license to publish the Supplemental Material files. Please contact the corresponding author directly for reuse.
