# Supplementary material for: Structural insights into manganese-dependent arylsulfatase from Enterococcus faecium and its catalytic promiscuity
Source: mBio. 2025 Aug 8;16(9):e00031-25. doi: 10.1128/mbio.00031-25 (PMC12421950; doi:10.1128/mbio.00031-25)
Supplement: Supplemental Material — Table S1 and Fig. S1 to S6. [file mbio.00031-25-s0001.pdf]

Supplementary Materials

Structural insights into manganese-dependent arylsulfatase from *Enterococcus faecium* and its catalytic promiscuity

Lulu Guo, Xuanjia Dong, Zetao Hu, Ling Zeng, Zhaohui Jin, Lin Jiang, Wenting Dai, Jinbiao Ma, Shili Chen, Ying Huang

Supplementary Table S1

Table S1 Information on various members of the alkaline phosphatase superfamily involved in the phylogenetic tree

Enzyme	Organism	accession number[a]	PDB ID	annotation[b]
PAS	Pseudomonas aeruginosa	P51691	1HDH	AS
BT4683_S1	Bacteroides thetaiotaomicron	Q89YP8	7ALL	AS
HARSB	Homo sapiens	P15848	1FSU	AS
HhGALNS	Hungatella hathewayi	A0A174CV66	6UST	AS
EcAS	Escherichia coli	A0A0H2V4H2	3ED4	AS
BT4631_S1	Bacteroides thetaiotaomicron	Q89YV0	7P26	AS
PsFucS1	Pseudoalteromonas sp.MB47	A0A8M0FGN6	7AJ0	AS
HARSA	Homo sapiens	P15289	1AUK	AS
HGALNS	Homo sapiens	P34059	4FDI	AS
HARSC	Homo sapiens	P08842	1P49	AS
HSTS	Homo sapiens	P08842	8EG3	AS
EfAS	Enterococcus faecium	I3U6E8		AS
SpAS1	Silicibacter pomeroyi	Q5LLA5	4UPI	AS
SpAS2	Silicibacter pomeroyi	Q5LMH0	4UPL	AS
SpPMH	Silicibacter pomeroyi	Q5LKJ1	4UPK	PMH
ArPMH	Agrobacterium radiobacter	B9JE48	4UPH	PMH
BcPMH	Burkholderia caryophylli	Q45087	2W8S	PMH
RlPMH	Rhizobium leguminosarum	Q1M964	2VQR	PMH
SmPPM	streptococcus mutans	Q8DTU0	3M7V	PPM
BcPPM	Bacillus cereus	Q818Z9	3M8W	PPM

HAP	Homo sapiens	P05187	1EW2	AP
RAP	Rattus norvegicus	P15693	4KJD	AP
EcAP	Escherichia coli	P00634	1AJA	AP
AbAP	Antarctic bacterium	Q9KWY4	2W5V	AP
CeiPGM	Caenorhabditis elegans	G5EFZ1	5KGL	iPGM
TbiPGM	Trypanosoma brucei brucei	Q38AH1	3NVL	iPGM
GsiPGM	Geobacillus stearothermophilus	Q9X519	1EJJ	iPGM
ENPP1	Homo sapiens	P22413	6WET	NPP
ENPP2	Rattus norvegicus	Q64610	2XR9	NPP
ENPP3	Homo sapiens	O14638	6C01	NPP
ENPP5	Mus musculus	Q9EQG7	5VEN	NPP
ENPP6	Mus musculus	Q8BGN3	5EGE	NPP
EciPGM	Escherichia coli	P62707	1E58	iPGM
BsAP	Bacillus subtilis	P42251	2YEQ	AP
SpPPM	Streptococcus pneumoniae	A0A0H2UPL4	2HIY	PPM

[a] The protein database at Uniprot (as of November 2024).

[b] The annotation of protein at Uniprot (as of November 2024). PMH, phosphonate monoester hydrolase; AS, arylsulfatase; AP, alkaline phosphatase; NPP, nucleotide pyrophosphatase/phosphodiesterase; iPGM, cofactor-independent phosphoglycerate mutase; PPM, phosphopentomutase.

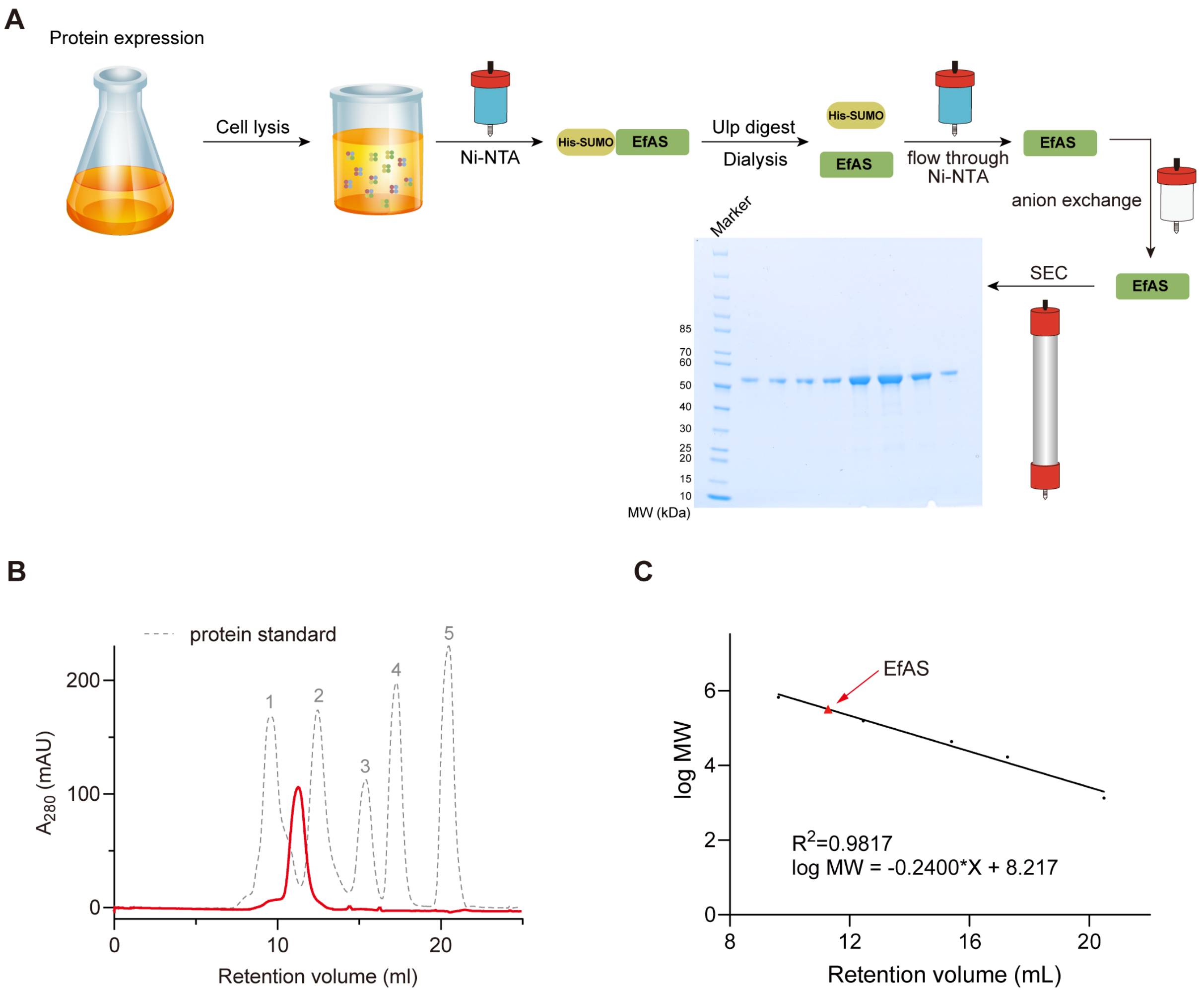

Figure S1. Purification of the EfAS. (A) Schematic of the purification steps. EfAS is first captured by Ni-affinity chromatography, followed by Ulp1 protease cleavage to remove the N-terminal His-SUMO tag. A second Ni-affinity step collects the flowthrough, leaving the cleaved tag bound. The protein is then purified by Q ion-exchange chromatography and finally by size-exclusion chromatography (SEC). The SDS-PAGE results of the SEC fractions are shown. (B) SEC elution profile from a Superdex 200 Increase 10/300 GL column, with dashed lines indicating protein molecular weight standards. (C) Standard curve used to estimate the apparent molecular weight of EfAS, which is indicated on the plot.

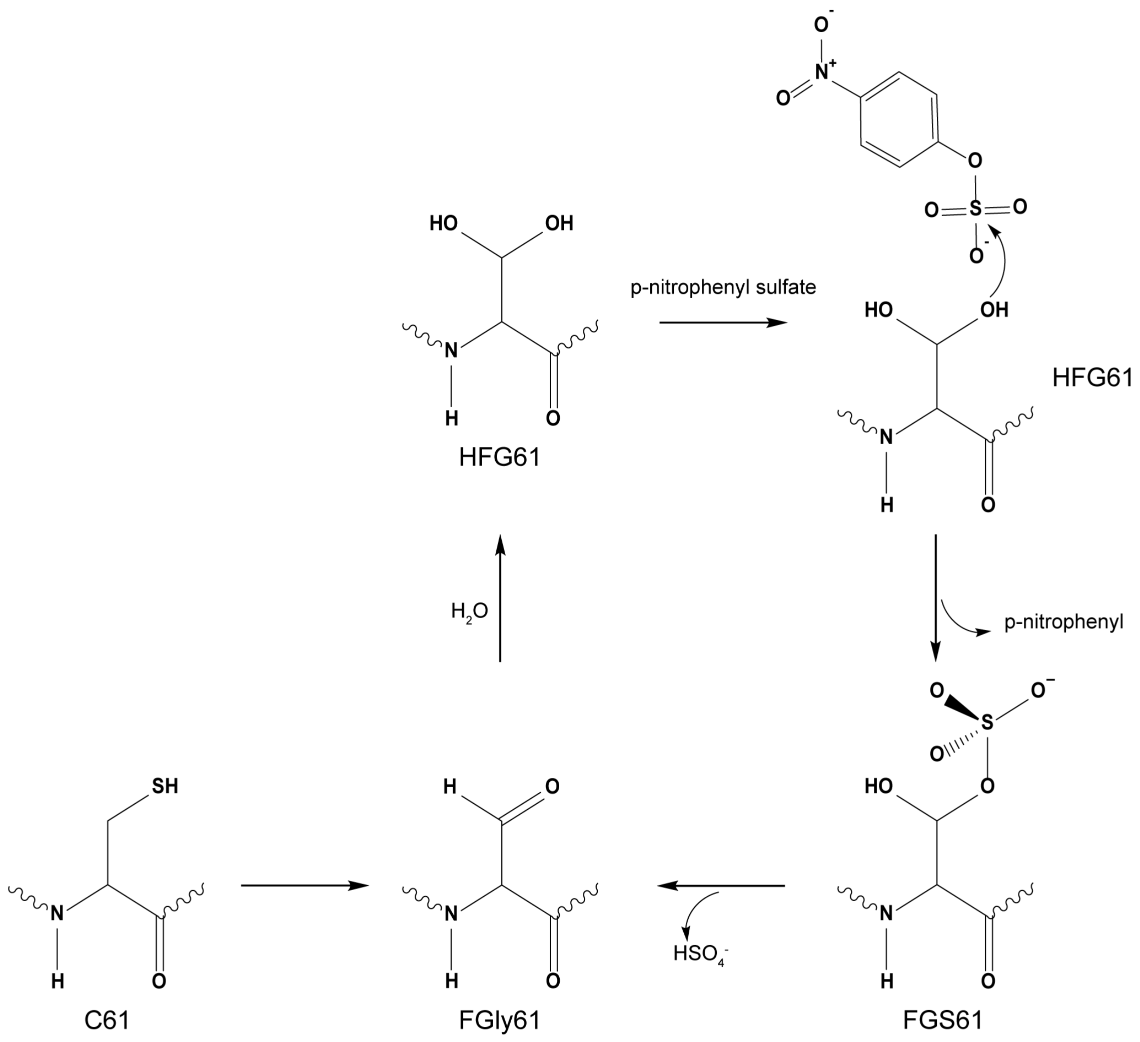

Figure S2. Schematic illustration of the proposed catalytic mechanism. EfAS removes sulfate groups from substrates, highlighting the key steps in the active site that facilitate bond cleavage and generation of the desulfated product.

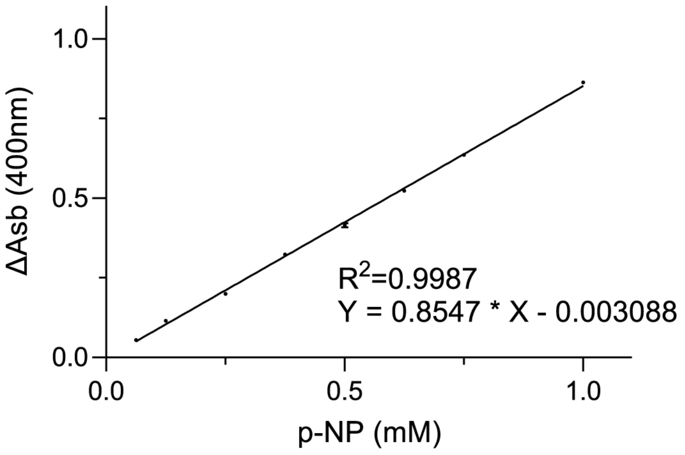

Figure S3. Establishment of a p-nitrophenol (pNP) concentration standard curve. pNP exhibits a distinct UV absorption peak at 400 nm under alkaline conditions. Standard samples were prepared at various concentrations, and their corresponding absorbance values at 400 nm were measured. The data were then fitted using GraphPad Prism software (version 9.5.0). Error bars represent SEM.

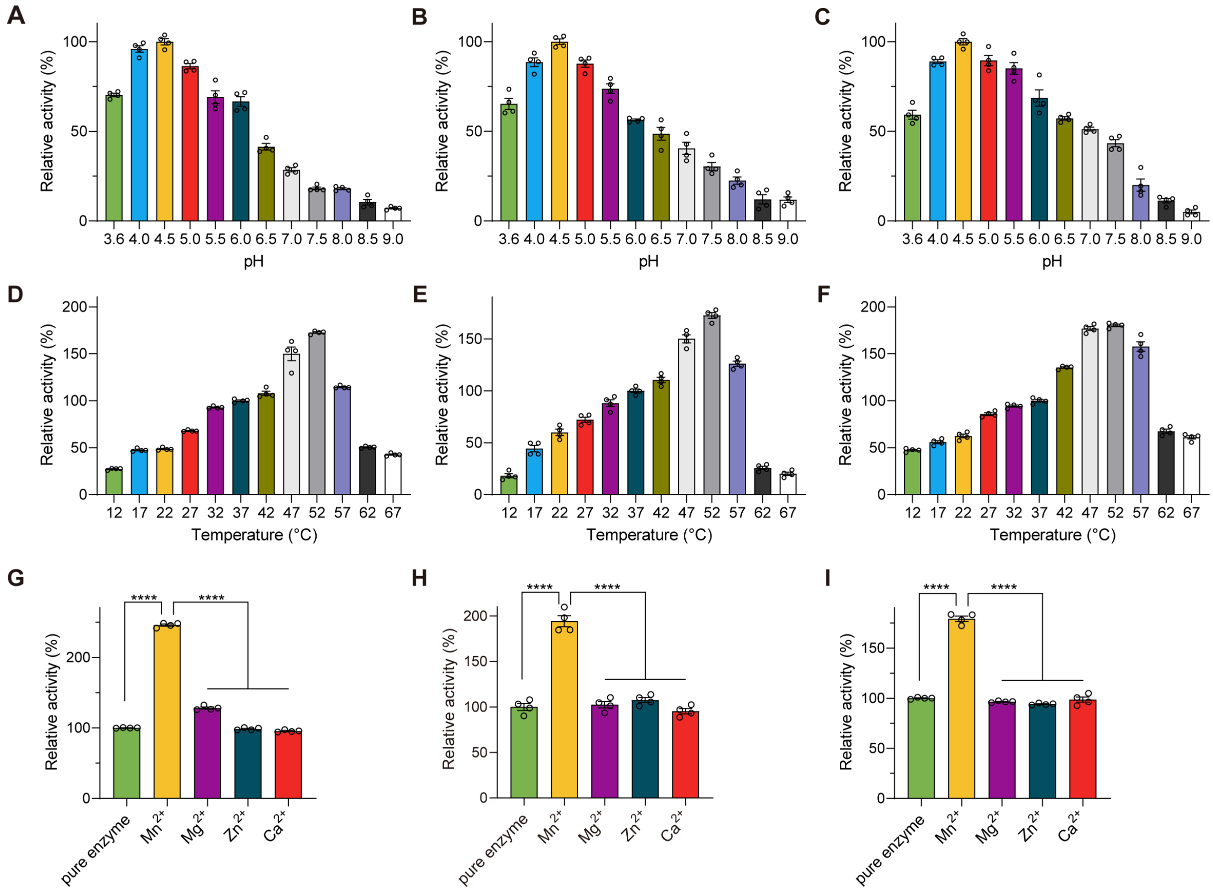

Figure S4. Enzymatic properties of EfAS under various conditions. (A-C) Enzyme activity was assessed under various pH conditions (3.6–9.0) following a 30-minute co-incubation at 37°C of EfASwith either 2 mM p-nitrophenyl phosphate (A), 2 mM p-nitrophenyl phenylphosphonate (B), or 1 mM p-nitrophenyl sulfate (C). (D-F) Enzyme activity was assessed under various temperature conditions (12-67°C) following a 30-minute co-incubation at pH 4.5 of EfAS with either 2 mM p-nitrophenyl phosphate (D), 2 mM p-nitrophenyl phenylphosphonate (E), or 1 mM p-nitrophenyl sulfate (F). (G-I) Enzyme activity was assessed under reaction conditions with 1 mM of various divalent metal ions following a 30-minute co-incubation at pH 4.5 and 37 °C of EfAS with either 2 mM p-nitrophenyl phosphate (G), 2 mM p-nitrophenyl phenylphosphonate (H), or 1 mM p-nitrophenyl sulfate (I).

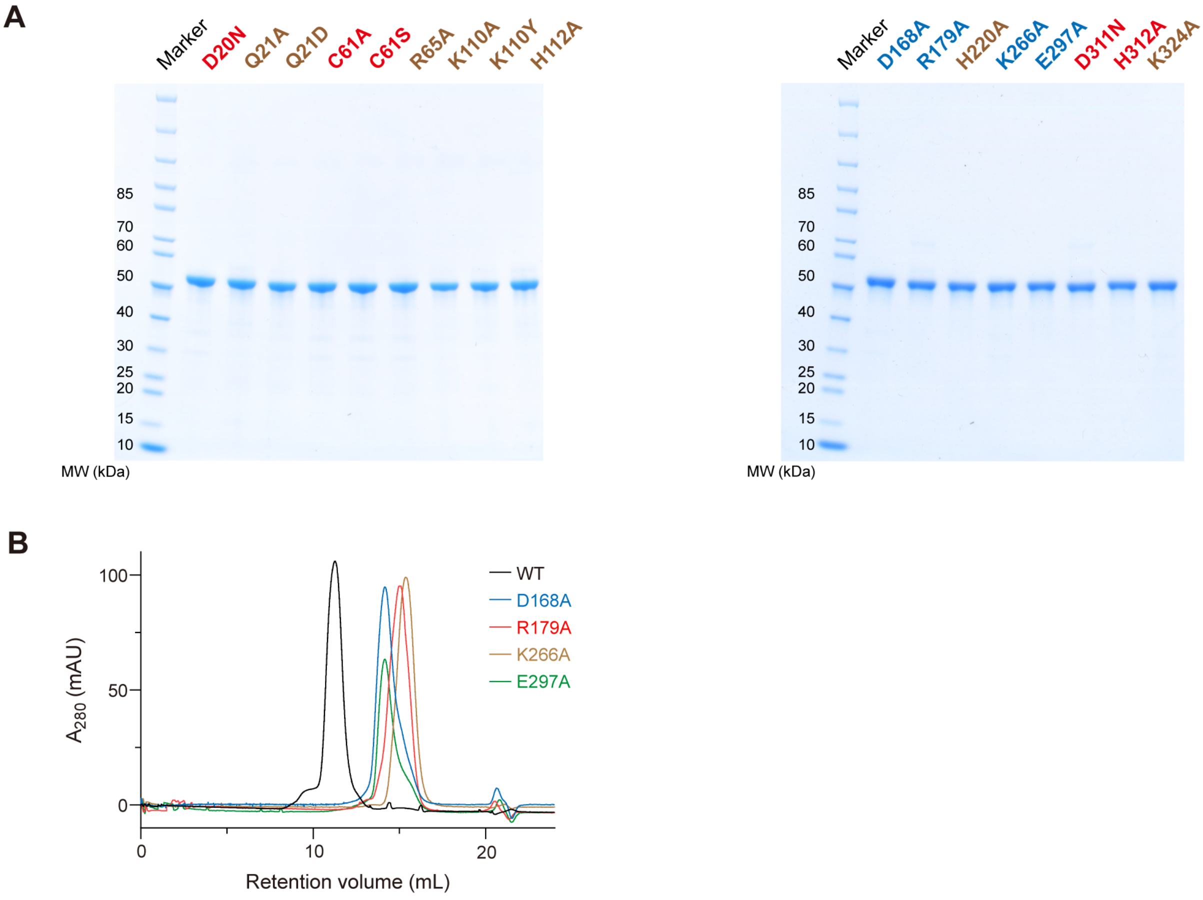

Figure S5. Purification and oligomeric state of EfAS mutants. (A) SDS–PAGE analysis showing the purity of mutant EfAS proteins. Mutants are color-coded by functional category: blue indicates tetrameric interface mutants, red indicates Mn^{2+} coordination site mutants, and brown indicates substrate-binding pocket mutants. (B) Size-exclusion chromatography elution profiles of the tetrameric interface mutants, which elute at later volumes compared to wild-type, consistent with a monomeric state.

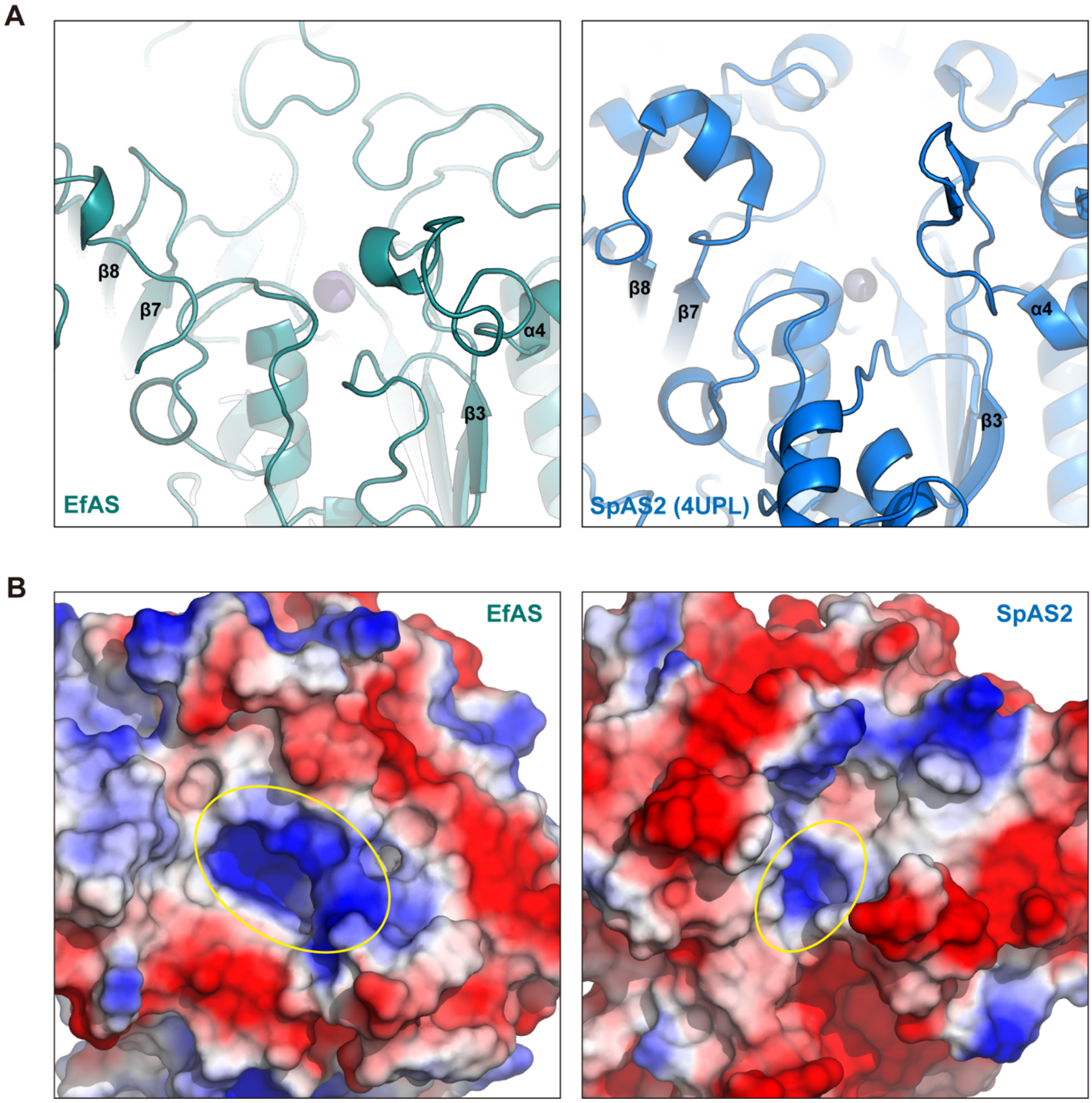

Figure S6. Structure comparison of EfAS and SpAS2. (A) Ribbon representation of the active-site regions of EfAS (left) and SpAS2 (right), highlighting the loops connecting β7-β8 and β3-α4, which contribute to differences in pocket architecture. (B) Electrostatic surface potential of the substrate-binding pockets in EfAS (left) and SpAS2 (right), showing distinct pocket shapes and charge distributions. Positively and negatively charged regions are colored in blue and red, respectively. Yellow circles indicate the substrate entry sites.
